# Potential use of nanoparticles produced from byproducts of drinking water industry in stabilizing arsenic in alkaline-contaminated soils

**DOI:** 10.1007/s10653-023-01663-z

**Published:** 2023-06-28

**Authors:** Mohamed L. Moharem, Hala M. Hamadeen, Mohamed O. Mesalem, Elsayed A. Elkhatib

**Affiliations:** 1 Regional Center for Food and Feed, Agricultural Research Center, Alexandria, Egypt; 2 Departments of Soil and Water Sciences, College of Agriculture (Elshatby), Alexandria University, Alexandria, 21545 Egypt

**Keywords:** Sorption isotherms, Kinetics, As speciation, Sequential extraction, Fourier transmission infrared spectroscopy

## Abstract

The stabilization of heavy metals in soils is considered a cost-effective and environmentally sustainable remediation approach. In the current study, the applicability of water treatment residual nanoparticles (nWTRs) with the particle size ranged from 45 to 96 nm was evaluated for its efficacy in reducing arsenic mobility in clayey and sandy contaminated alkaline soils. Sorption isotherms, kinetics, speciation and fractionation studies were performed. Sorption equilibrium and kinetics studies revealed that As sorption by nWTRs-amended soils followed Langmuir and second-order/power function models. The maximum As sorption capacity (q_max_) of Langmuir increased up to 21- and 15-folds in clayey and sandy soils, respectively, as a result of nWTRs application at 0.3% rate. A drastic reduction in non-residual (NORS) As fraction from 80.2 and 51.49% to 11.25 and 14.42% for clayey and sandy soils, respectively, at 0.3% nWTRs application rate was observed, whereas residual (RS) As fraction in both studied soils strongly increased following nWTRs application. The decline in percentage of As mobile form (arsenious acid) in both soils after nWTRs application indicated the strong effect of nWTRs on As immobilization in contaminated soils. Furthermore, Fourier transmission infrared spectroscopy analysis suggested reaction mechanisms between As and the surfaces of amorphous Fe and Al oxides of nWTRs through OH groups. This study highlights the effective management approach of using nWTRs as soil amendment to stabilize As in contaminated alkaline soils.

**Supplementary Information:**

The online version contains supplementary material available at 10.1007/s10653-023-01663-z.

## Introduction

Natural and anthropogenic activities such as coal and ore mining and use of arsenical compounds in agriculture contributed largely to arsenic (As) contaminations of agricultural soils (Dubey et al., 2022; Mandal & Suzuki, 2002; Smith et al., 1998). In alkaline agricultural soils, solubility of As enhances and its high leaching potential through soil profile triggers contamination of groundwater and surface waters that present a constant danger to population health (Niazi et al., 2011; Patel et al., 2022; Smith & Steinmaus, 2009; Yuan et al., 2007).

Water treatment residuals (WTRs), byproducts of drinking water treatment industry, are counted one of the widespread aluminum- and iron-rich wastes. These daily generated wastes in the course of water treatment process have been frequently used as an effective strategy for remediation of heavy metals polluted soils (Elkhatib et al., 2013; Elkhatib & Moharem, 2015; Sarkar et al., 2007a). Because of the instability of the arsenic species, different soil environments and components such as aluminum-iron oxides, organic matter and carbonates may greatly affect As behavior. Thus, the effective restoration of As-polluted soils requires sound knowledge of As interaction with soil components. Several researchers have examined the potential of WTRs in restoring As-polluted soils (Nielsen et al., 2011; Rathnayake & Schwab, 2022; Sarkar et al., 2007a, 2007b). They found that utilization of Fe WTRs at a rate of 3% successfully stabilized heavily polluted soils with heavy metals including arsenic (Garau et al., 2014). Nagar et al., (2014, 2015) emphasized the successful use of Fe and Al WTRs in reducing the bioaccessible As in two polluted soils.

Recently, the nanostructured water treatment residuals (nWTRs) have been developed and proved to outperform the bulk water treatment residuals (WTRs). The remarkable characteristics of nWTRs like large surface area, high retention capacity and stability are useful in remediation of As-polluted soil and water (Elkhatib et al., 2015a). Because the conventional bulk WTRs have restrained reactivity with regard to pollutants, Elkhatib et al. (2015a) pioneered the production and use of nWTRs for soil and water remediation. Several retention studies exhibited that the capacity of nanostructured WTRs in retaining As, Hg, Cd, Cr and P from contaminated wastewater much exceeded that of bulk WTRs by 16.7,13, 16.8, 15 and 30 times, respectively (Elkhatib & Moharem, 2015; Elkhatib et al., 2016, 2019; Hamadeen et al., 2022). Therefore, we have hypothesized that producing nanostructured super sorbent using the inexpensive byproducts of water industry (WTRs) would markedly magnify the capability of bulk counterpart for remediation of As-contaminated soils. Additionally, application of nWTRs as a novel solution to generate ecofriendly nano-sorbents from waste materials would demonstrate a clear-cut role in recycling waste supporting environmental-friendly practices (Keeley et al., 2014; Ren et al., 2020).

Information concerning the use of nanoparticles in remediating the As-contaminated high pH soils is scarce. Therefore, the purposes of the present investigation were to: (1) determine the capability of nWTRs to stabilize As under alkaline conditions of two selected arid soils, (2) fractionate As among different soil components of the soils received various nWTRs rates, (3) study the effect of nWTRs on As species in nWTRs-treated alkaline soils using chemical equilibrium calculations (MINEQL + 4.6 model), (4) elucidate the adsorption mechanism of As(V) onto nWTRs-applied soils using sorption equilibrium and kinetics data and FTIR spectroscopic analysis.

## Materials and methods

### Soils and WTRs

Two soils representative of arid area, a sandy soil (Typic torripsamment) and a clayey soil (Typic Torrifluvents), were collected from Elbostan-Alexandria (30º 54′ N, 29º 52′ E) (Fig. S1-Supplementary Information) and Kafr El-Dawar-Elbohera (31º 13′ N, 30º 25′ E) (Fig. S2-Supplementary Information), respectively. The soil samples were air-dried and 2 mm sieved. The WTRs samples were collected from Kafr El-Dawar drinking water treatment facilities (31º 08′ N, 30º 08′ E), Fig (S3-Supplementary Information), air-dried and sieved (< 2 mm). Soils and WTR chemo-physical properties were determined according to standard methods described by Page (1982) (Table1). The nanoparticles of WTRs were produced by milling WTRs-subsamples employing the method of Elkhatib et al. (2015b).

**Table 1:** Selected physical and chemical characteristics of studied soils and drinking water treatment residuals. OM, organic matter; SL, sandy loam; nd: not determined; WTRs, water treatment residuals

Characteristics	Units	Typic torrifluvent ^a^	Typic torripsamment	WTRs
pH		8.13 ± 0.05	7.69 ± 0.05	7.45 ± 0.06
EC	dSm^−1^	2.66 ± 0.11	3.84 ± 0.12	1.67 ± 0.04
CaCO_3_	g kg^−1^	57.90 ± 0.60	2.40 ± 0.30	nd
Sand	g kg^−1^	596.4 ± 4.20	868.2 ± 5.10	nd
Silt	g kg^−1^	141.3 ± 1.50	25.10 ± 0.30	nd
Clay	g kg^−1^	262.30 ± 3.70	106.70 ± 2.20	nd
Texture		SCL	LS	nd
O.M	g kg^−1^	8.50 ± 0.15	1.00 ± 0.04	57.00 ± 2.00
KCl-Al	mg kg^−1^	1.03 ± 0.04	0.13 ± 0.02	28.18 ± 1.03
Olsen-P	mg kg^−1^	24.75 ± 0.25	2.89 ± 0.14	24.00 ± 2.00
CEC	Cmol (+) kg^−1^	39.13 ± 0.98	8.70 ± 0.20	34.78 ± 0.34
Total As	mg kg^−1^	1.66 ± 0.13	1.23 ± 0.11	1.04 ± 0.02

OM , organic matter ; SL, sandy loam; nd: not determined; WTRs, water treatment residuals

^a^Means of three samples ± SD

## Characterization of nWTRs

Scanning electronic microscope with energy-dispersive X-ray (SEM–EDX; INCAx-Sight model 6587, Oxford Instruments, UK) with magnifications of × 100 to × 5000 with multiple images captured and transmission electron microscopy (TEM, CM200, Phillips) were used to examine the surface features, size and elemental contents of nWTRs, and X-ray diffraction (XRD, PW1710, Philips, Holand) was used for the primary characterization of material properties. The surfaces chemistry of nWTRs surfaces was investigated using Fourier transform infrared spectroscopy (FTIR, Model Perkin Elmer 400, USA), and surface area of the material was determined using surface area analyzer (Quantachrome, USA) using N_2_ gas adsorption/desorption at 77 K. (Hamadeen et al., 2021).

## Application rate of nWTRs

The two studied soils were amended with three ratios of nWTRs (0, 0.1, 0.2 and 0.3% w/w DW) and a one ratio (2%) of bulk WTRs. The control has no additions of WTRs. The soils and nWTRs or WTRs samples were fully mixed and transmitted to big plastic boxes. The moisture contents of the nWTRs-amended and non-amended soils were adjusted to field capacity using deionized water. The moisture contents of the amended and non-amended soils were preserved stable during the 30 days incubation period at 25 °C. Then, the soils were air-dried, 2 mm sieved and divided in sub-samples for the adsorption studies.

## Arsenic sorption isotherms

Batch sorption equilibration study was conducted in order to examine As sorption by the nWTRs/ WTRs**-**treated soils. The As sorption study was performed using 20 mL of Na_2_HAsO_4_.7H_2_O with initial concentrations of 5, 10, 20, 40, 80 and 160 mg As L^−1^. Arsenate solutions were added to the unamended and WTRs/nWTRs-amended soil samples in a 50-mL centrifuge tubes. The mixture was equilibrated for 24 h at 20 ± 3 °C, centrifuged for 10 min at 5000 rpm and filtered. Then, 10 mL of the supernatant solution was used for As determination by atomic absorption spectrometry (contr AA 300, Hydride unit). The amount of sorbed As was calculated as the difference between the initial and final solution concentrations. All measurements were executed in triplicate.

## Adsorption kinetics

Kinetic experiments were run in a closed system to display the effect of contact time on arsenate sorption onto untreated and/or nWTRs-/WTRs**-**treated soils at room temperature (22 ± 2 °C). The untreated or treated soil samples with the initial As concentration of 160 mg L^−1^ were placed in 50-mL centrifuge tubes and agitated for different time intervals (from 15 to 1440 min). Centrifugation, analysis and calculation were run as previously described in sorption experiments.

## Arsenic fractionation

Heavy metals (HMs) fractionation is an important process regarding environmental and remediation studies since it provides useful information about the fate/behavior of HMs in contaminated sites via mobility, fixation, bioavailability and plant uptake. The collected clay and sandy soil samples, originally contained 1.66 and 1.23 mg As kg^−1^, were treated with 300 mg As kg^−1^ (Na_2_HAsO_4_.7H_2_O), and fractionation of soil As was undertaken using the sequential extraction procedure (Tessier et al., 1979). In this technique, the distribution of As within various components of contaminated soils before and after nWTRs application rates was determined. Briefly, this procedure assorted soil As into the following categories: exchangeable, carbonates, Fe–Mn oxides, OM and residual (RS). All the analyses were executed in triplicates, and As concentration in each extract was measured using atomic absorption (contrAA 300, hydride unit).

## Arsenic speciation

Arsenic toxicity/mobility in soil environment is greatly affected by the oxidation state and ionic species of As in soil solution. Arsenic species in contaminated soils before and after nWTRs addition were calculated using the MINEQL + 4.6 program which is specifically designed for calculation of metal species in contaminated soils (Schecher & McAvoy, 2007). This program is run using pCO_2_, pO_2_, pE, electric conductivities, calculated ionic strength, pH, elements concentration in soil solutions of the nWTRs-/WTRs-treated and untreated soils. The soil solution samples were acquired using the rapid centrifugation method of Elkhatib et al. (1987).

## Results and discussions

### Characterization of nWTRs

**Fig. 1:**
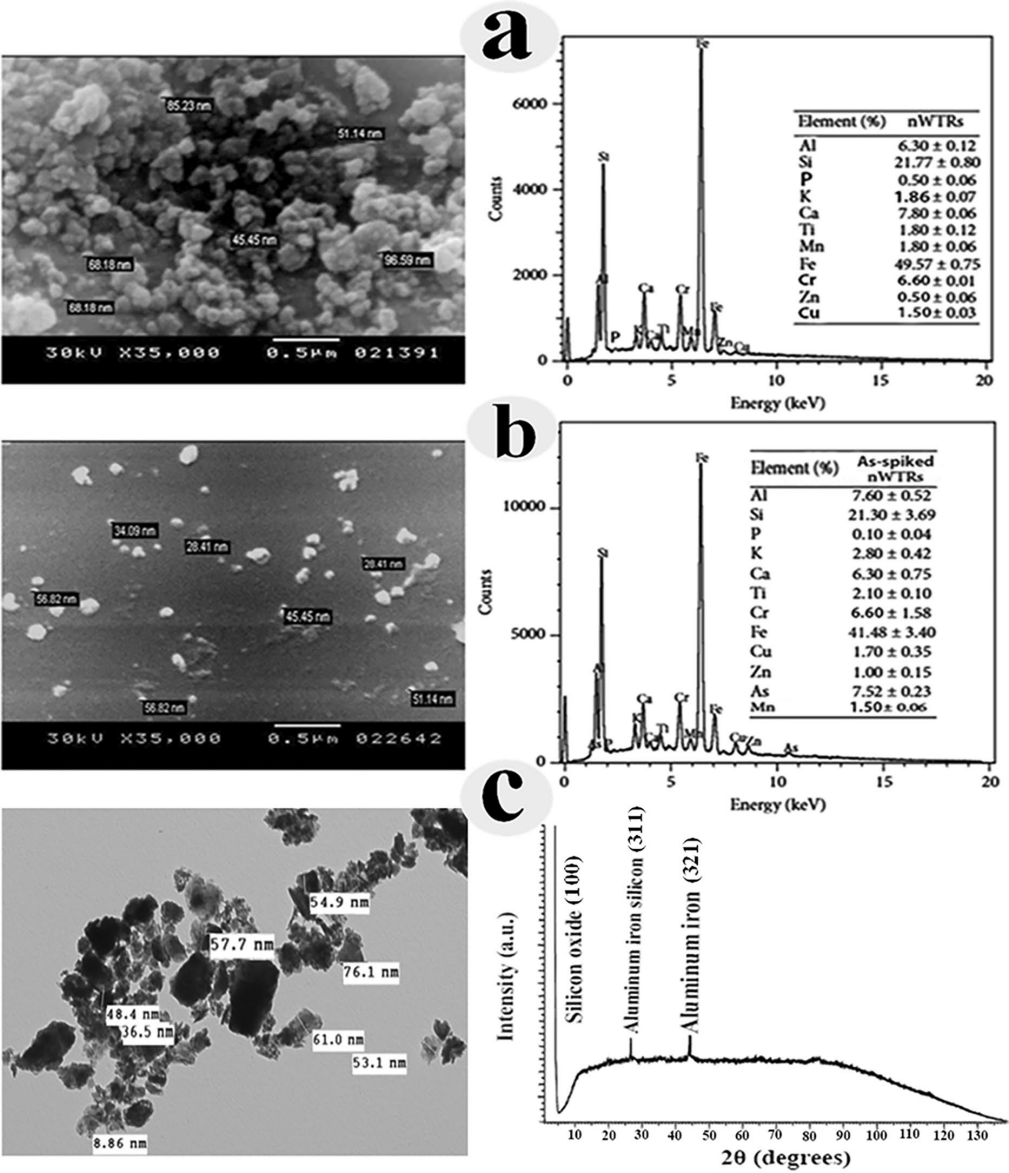
Scanning electron microscopy (SEM) image and energy-dispersive X-ray (EDX) spectrum of **a** nWTRs, **b** the As-spiked nWTRs, **c** transmission electron microscopy (TEM) image (left) and X-ray diffraction analysis (right) of nWTRs

The SEM image of nWTRs (Fig. 1a) showed a spherical morphology of WTRs nanoparticles with different sizes in the nanoscale range (45 to 96 nm). The EDX-spectrum of nWTRs revealed domination of iron, silicon, calcium and aluminum oxides (Elkhatib et al., 2015a). The SEM image of As loaded nWTRs (Fig. 1b-left) exhibited a covering layer of As on nWTRs surface which is confirmed by the presence of high percentage of As (7.52%) in As-loaded nWTRs relative to nWTRs alone (Fig. 1b-right).

The TEM image of the nWTRs (Fig. 1c- left) showed that the WTR nanoparticles are in somewhat agglomerated state and the particle sizes fall in the nanoscale within the range of 8.9–76.1 nm.

The X-ray diffraction (XRD) pattern (Fig. 1c- right) ascertained that amorphous iron aluminum (hydr)oxides and silicon oxide dominated all nWTR, with no apparent crystalline iron–Al(hydr)oxides. The abundant iron and aluminum in water treatment residual nanoparticles could have great influence on As stabilization in nWTRs-amended soils.

BET-specific surface area and the total pore volume of WTRs and nWTRs samples were determined. The WTRs have a specific surface area of 53.1 m^2^ g^−1^ and have a total pore volume of 0. 020 cm^3^ g^−1^ while nWTRs have a specific surface area of 129 m^2^ g^−1^ and have a total pore volume of 0. 051 cm^3^ g^−1^. The specific surface area of nanoscale WTRs sample is 2–3 times higher than that of WTRs.

## Arsenic sorption isotherms

**Fig. 2:**
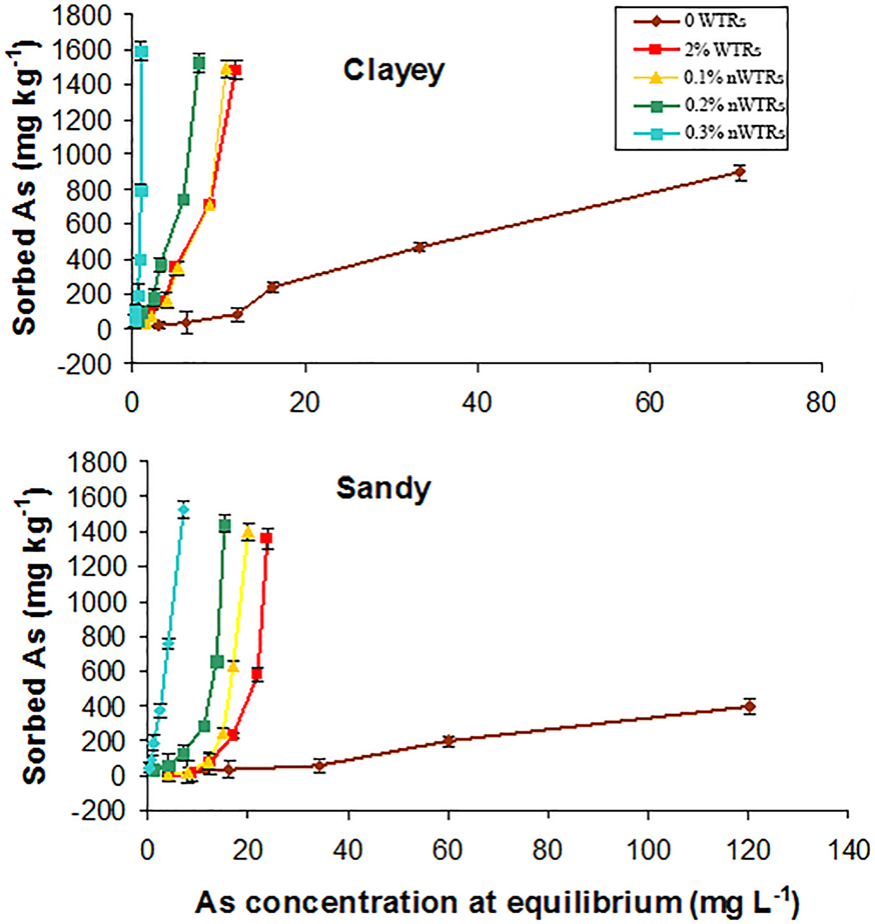
Arsenic sorption isotherms for the two studied soils as affected by different rates application of drinking water treatment residual nanoparticles (nWTRs). WTRs, water treatment residuals

Sorption isotherms of As for studied soils prior to and following WTRs**/**nWTRs application are illustrated in Fig. 2. The sorption isotherms of the un-amended soils (control) showed low As sorption capacity with lesser affinity for sandy soil. According to Giles et al. (1960), both sorption isotherms were L-type isotherms which indicate low concentration of adsorbate and low affinity of sorbent for the adsorbate. The slightly higher amount of As sorbed in clayey soil could be referred to the higher CEC of clayey soil (Table 1). Sahoo and Kim (2013) demonstrated that in clay-rich soils, As(III) and As(V) sorption may be encouraged due to the easily incorporation of FeOOH in the clay size soil fraction. However, the studied soils, including clayey soil, exhibited low affinity for As retention (Fig. 2) due to the alkaline conditions of the soils studied (Table1). Many authors reported the increasing mobility and release of As(III) and As(V) under alkaline conditions (DeMarco et al., 2003; Lewińska et al., 2018; Nagar et al., 2010).

Amending both soils with bulk and nanoparticles of WTRs increased soils capability for retaining arsenic with nanoparticles being most effective. The S-type adsorption isotherms of the studied soils were converted to H-type at a very low nWTRs application rate (0.1%) indicating high sorption affinity of nWTRs-amended soils for As (Fig. 2). Such dramatic increases in As retention by the nWTRs-amended soils could be due to the high specific surface area of nWTRs (129 m^2^ g^−1^) which supplied both studied soils with supremely effective sorption sites (Elkhatib et al., 2015c).

## Modeling As sorption equilibrium

Sorption equilibrium models are intrinsic equations that relate concentrations of adsorbate in the solid phase (adsorbent) and the adsorbate concentrations remaining in the liquid phase at equilibrium. The calculated parameters of the sorption models furnish important information related to surface reactivity and sorbent–sorbate interactions (Elkhatib et al., 2017). Four sorption equilibrium models (Langmuir, Freundlich, Elovich and Temkin) were used to characterize As sorption by nWTRs-/WTRs-amended and non-amended soils. The models and its calculated parameters are presented in Table 2 and Table (S1). Based on high *R*^2^ (coefficient of determination) and low SE (standard error) values of the models tested, Langmuir isotherm model best described As adsorption by the studied soils (Table 2). The better fit of As adsorption data to Langmuir model suggests the monolayer coverage of As on soils surface (Elkhatib et al., 2017). The estimated Langmuir parameters *q*_max_ and *K*_*L*_ (Table 2) represent the maximum adsorption capacity of the sorbent for As (μgg^−1^) and affinity constant, respectively. It can be seen from Table 2 that application of nWTRs greatly enhanced the *q*_max_ of both clayey and sandy soils. Application of nWTRs at the lowest rate of 0.1% increased *q*_max_ of clayey and sandy soils by 6.6 and 3.4 times, respectively. Moreover, increasing application rate of nWTRs to 0.3% increased *q*_max of_ clayey and sandy soils by 21.8 and 14.9 times, respectively. Similarly, the *K*_*L*_ values of Langmuir model considerably increase with nWTRs application reflecting increase in As affinity toward nWTRs. These results evidently demonstrate the potential benefits of nWTRs application in As stabilization in contaminated alkaline soils.

**Table 2:** Langmuir and Fruendlich isotherms parameters for As sorption onto the studied soils as affected by WTRs/nWTRs treatments

Treatment	Freundlich				Langmuir			
	*q*_*e*_ = *K*_*F*_ *C*_*e*_^1/*n*^				1/*q*_*e*_ = (1/*K*_*L*_ *q*_max_)1/**C**_**e**_ + 1/q_max_			
	*K*_*F*_ (mL g^−1^)	1/*n*	*R*^2^	SE	*q*_max_ (μg g^−1^)	*K*_*L*_ (L mg^−1^)	*R*^2^	SE
*Clayey soil*								
0WTRs	0.611	1.31	0.969	0.1266	153.12	0.018	0.991	0.0021
2%WTRs	1.3206	1.6765	0.992	0.0567	989.43	0.024	0.965	0.0024
0.1%nWTRs	1.1777	1.8455	0.9843	0.0855	1012.31	0.029	0.939	0.0030
0.2% nWTRs	1.8919	1.3246	0.9751	0.1016	1981.51	0.037	0.990	0.0030
0.3% nWTRs	2.8418	1.378	0.7838	0.2948	3333.33	0.130	0.920	0.0053
*Sandy soil*								
0WTRs	0.2206	1.1206	0.978	0.1031	95.89	0.003	0.997	0.0024
2%WTRs	1.4804	3.1611	0.917	0.2690	295.31	0.009	0.944	0.0162
0.1%nWTRs	1.1366	3.0765	0.893	0.3085	321.52	0.026	0.976	0.0068
0.2% nWTRs	1.2434	1.3121	0.819	0.2897	588.24	0.053	0.864	0.0042
0.3% nWTRs	2.2123	1.0744	0.985	0.0760	1428.57	0.127	0.973	0.0016

R^2^, determination coefficient; SE, standard error of estimate; WTRs, water treatment residuals; nWTRs, water treatment residual nanoparticles

## Arsenic sorption kinetics

**Fig. 3:**
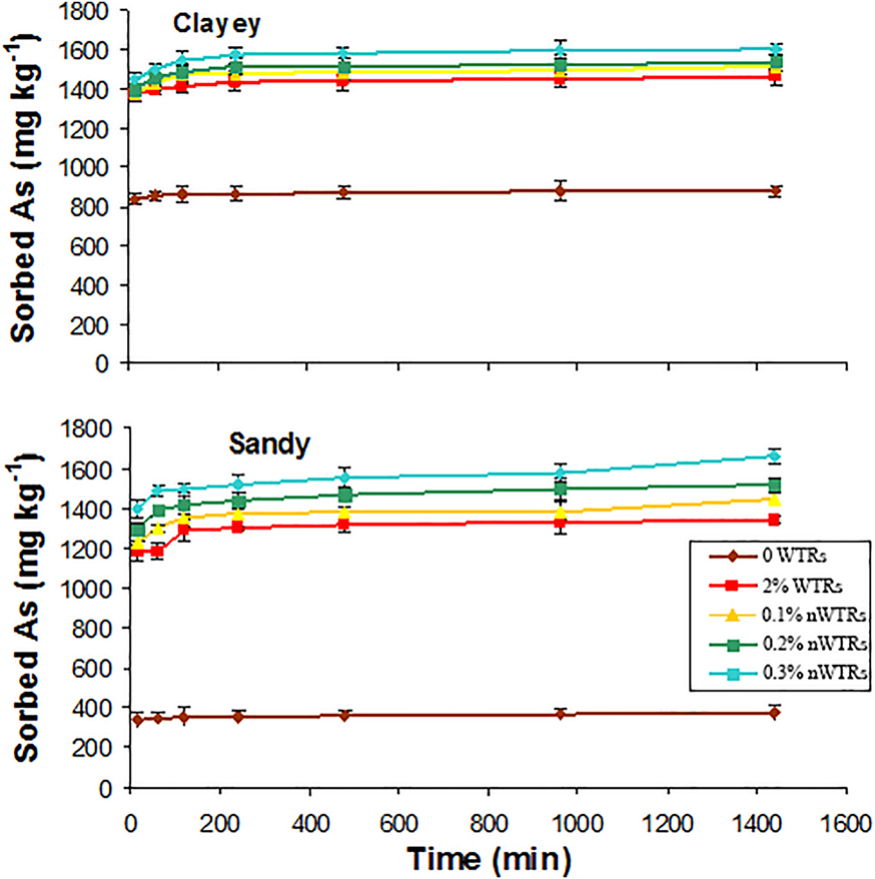
Arsenic sorption kinetics for the two studied soils as affected by different application rates of drinking water treatment residual nanoparticles (nWTRs). WTRs, water treatment residuals

The effect of contact time (15–1440 min) on As sorption onto un-amended or nWTRs-/WTRs**-**amended clayey and sandy soils was studied, and the results are presented in Fig. 3. Sorption kinetics of As by the nWTRs-/WTRs-amended and non-amended soils were biphasic with fast sorption reaction followed by a considerably slower sorption reaction before achieving equilibrium. Within the first 60 min, a very fast sorption reaction occurred and almost 95% of total As in solution was removed. With the increase in contact time, the sorption reaction got slower with only 2–5% of total As was removed until equilibrium was reached. The obtained results are in agreement with the previous studies on adsorption of arsenic species by composite sorbents and natural heterogeneous solids surfaces (Cheng et al., 2015; Hafeznezami et al., 2016; Neupane et al., 2014; Smith & Naidu, 2009). Applications of nWTRs at a high rate (0.3%) to both soils studied have led to 77% increase in the sorption capacity of both soils compared with control soil. The strong reaction of amorphous Fe and Al in nWTRs through inner-sphere complexion could be responsible for the high As sorption capacity of nWTRs-amended soils (Goldberg & Johnston, 2001; Manning et al., 1998; Sherman & Randall, 2003; Zhang & Selim, 2005).

## Modeling As sorption kinetics

**Fig. 4:**
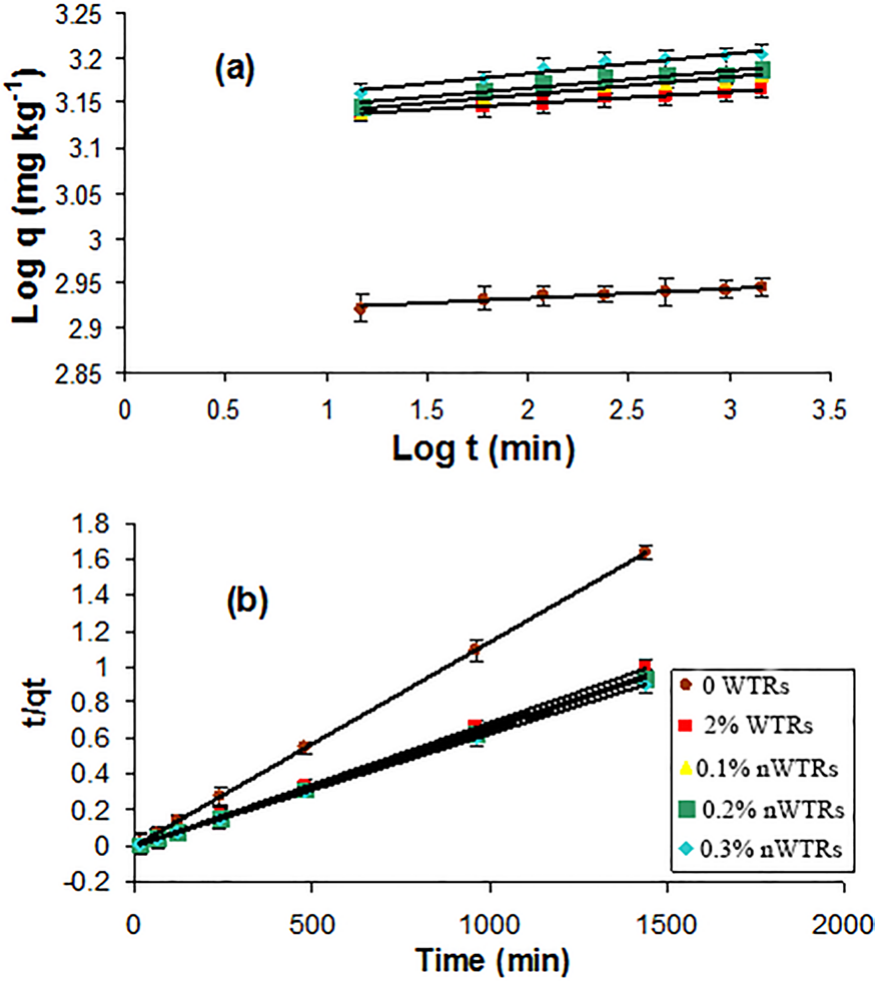
Power function **a** and second-order **b** model plots for As sorption by clayey soil amended with different rates of drinking water treatment residual nanoparticles (nWTRs). WTRs, water treatment residuals

Sorption kinetic data of arsenic by nWTRs-amended clayey and sandy soils were modeled using the first-order, second-order, parabolic diffusion and power function models (Elkhatib & Hern, 1988). Range and mean values of coefficient of determination (*R*^2^) and standard error of estimates (SE) for the four kinetic models fitted to As sorption kinetics on the studied soils amended with or without WTRs/nWTRs are presented in Table 3. The parabolic diffusion model was not appropriate to depict all the kinetic sorption data due to its low R^2^ and high SE values (*p* ≥ 0.05) (Table 3). Similarly, the first-order model didn't fit to the kinetic sorption data as shown from the relatively high SE and the low (*R*^2^) values. However, the sorption kinetic data for As were best described by power function model followed by second-order kinetic model due to their high *R*^2^ and low SE values, which suggests the role of chemo-sorption reaction onto nWTRs surfaces in controlling the reaction-determining step (Elkhatib et al., 2015c; Feng et al., 2009; Liu et al., 2012). The kinetics of As sorption during sediment resuspension was also found to follow second-order model (Wang et al., 2018). The linear plots of the power function and the second-order models are shown in Fig. 4, and the kinetic model parameters obtained from the slope and intercept of linear plot of both models are given in Table 4.

**Table 3:** Range and mean values of coefficient of determination (*R*^2^) and standard error of estimates (SE) for different kinetic models fitted to As sorption kinetics on the two studied soils amended with or without WTRs/nWTRs

Model	*R*^2^			SE		
	Range			Range		
	Min	Max	Mean	Min	Max	Mean
First-order kinetic	0.487	0.981	0.787	0.0836	1.1818	0.4208
Intraparticle diffusion	0.989	0.814	0.814	1.1675	68.4539	26.2849
Power function	0.861	0.990	0.935	0.0007	0.0206	0.0059
Second-order kinetic	0.998	1.000	0.999	0.0011	0.0202	0.0062

WTRs, water treatment residuals; nWTRs, water treatment residual nanoparticles

The sorption rate constant (*k*_*a*_) of power function model well expressed the influence of nWTRs application on As sorption rate in the clayey and sandy soils. The sorption rate constants increased with increasing nWTRs application rate (Table 4). At 0.3% nWTRs application rate, the *k*_*a*_ value increased from 825 to 1377 min^−1^ in clayey soil and from 325 to 1249 min^−1^ in sandy soil. This suggests that As adsorption onto nWTR amended soils is governed by chemical forces.

**Table 4:** Power function and second-order parameters of As sorption onto the two studied soils as affected by WTRs/nWTRs treatments

Treatment	Power function		Second-order	
	*q* = *k*_*a*_ *C*_*o*_ *t*^1*/m*^		*t*/*q*_*t*_ = 1/*k*_2_*q*_*e*_^2^ + t/q_e_	
	*k*_*a*_ min^−1^	1/*m*	q_e_ (μg g^−1^)	k_2_ (gμ g^−1^ min^−1^)
*Clay soil*				
0WTRs	825	0.009	909	3.8 × 10^–4^
2%WTRs	1311	0.0134	1418	1.8 × 10^–4^
0.1% nWTRs	1324	0.0188	1428	1.6 × 10^–4^
0.2% nWTRs	1338	0.0199	1448	1.8 × 10^–4^
0.3% nWTRs	1377	0.0219	1666	1.4 × 10^–4^
*Sandy soil*				
0WTRs	325	0.0166	370	3.4 × 10^–4^
2%WTRs	1078	0.0315	1325	9.6 × 10^–5^
0.1%nWTRs	1141	0.0316	1328	6.3 × 10^–5^
0.2% nWTRs	1198	0.0331	1399	7.7 × 10^–5^
0.3% nWTRs	1249	0.0368	1548	4.1 × 10^–5^

WTRs, water treatment residuals; nWTRs, water treatment residual nanoparticles

## Arsenic fractionation

**Fig. 5:**
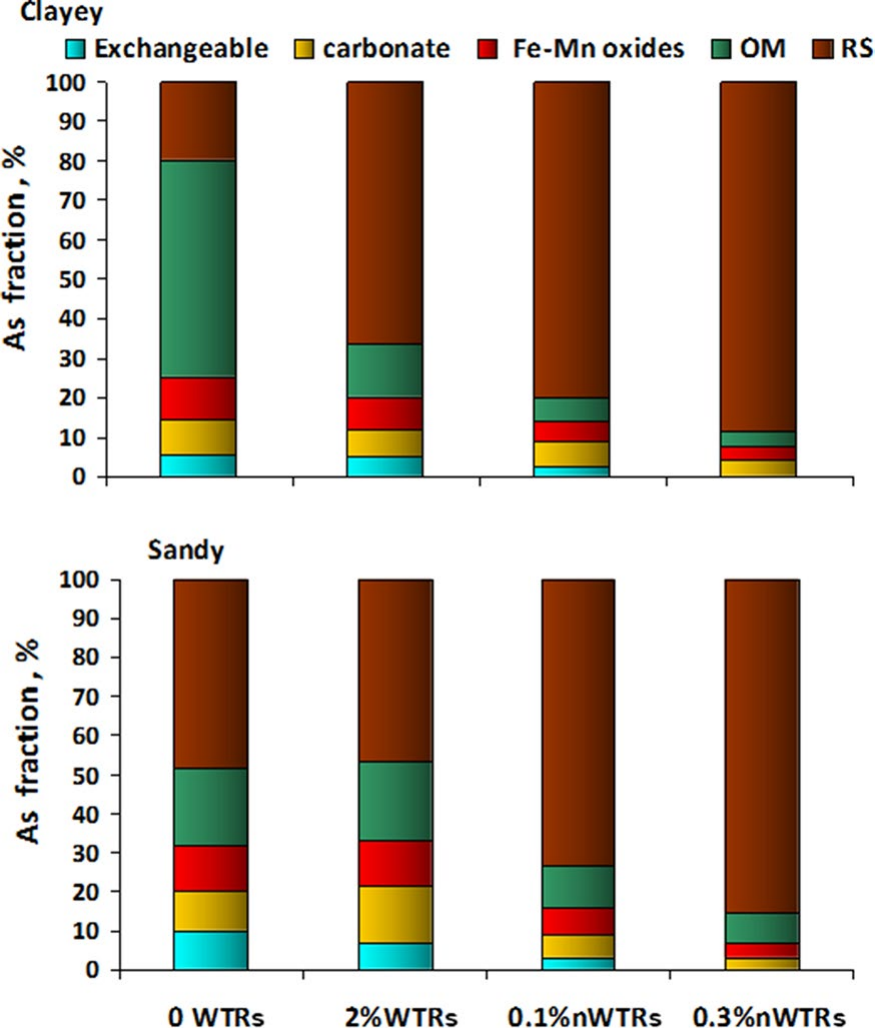
Relative percentage of As fractions for the two contaminated soils amended with WTRs at a rate of 2% or nWTRs at rates of 0.1 and 0.3% by weight. WTRs, water treatment residual; nWTRs, water treatment residual nanoparticles; RS, residual fraction; OM, organic matter

Chemical pattern of the metal greatly governed its fate and behavior in soil environment. A fractionation technique of Tessier et al. (1979) was utilized to examine As distribution in different lattices of the soil–solid samples. Based on this technique, soils arsenic was fractionated into five chemical fractions following the order of decreasing solubility in the sequence: exchangeable > carbonate > oxides > organic > residual (Schramel et al., 2000). The changes of As geochemical forms in unamended and amended contaminated clayey and sandy soil samples with WTRs/nWTRs are depicted in Fig. 5. In un-amended contaminated soils, the dominant percentage of As in clayey soil was in the organic fraction (55%) followed by residual fraction (19.8%) and the exchangeable fraction was the least fraction occupied by As (5.4%). In sandy soil, the highest percentage of As was present in the residual fraction (48.5%) while the least presence of As was in the exchangeable fraction (9.9%). The organic fraction of clayey soil exhibited higher percentage of As (55%) than that of the corresponding sandy soil (22.77) which could be attributed to the higher amount of organic matter (OM) of clayey soil than sandy soil (Table 1). In addition, more As percent was existed in the sandy exchangeable fraction (9.9%) than corresponding clayey soil (5.4%). These results indicate that As in sandy soil is weakly sorbed and more mobile than in clayey soil which could be referred to the low clay and OM contents in sandy soil than in clayey soil (Table 1). Other researchers also found that As was much more mobile in sandy soil than in OM-/clay-rich soil (Datta et al., 2006; Nagar et al., 2014; Quazi et al., 2011; Wang & Mulligan, 2006). Bhattacharya et al. (2013) reported strong correlation of distribution pattern of arsenic in soil with oxidizable organic carbon content of soil.

Application of nWTRs significantly changed the distribution pattern of As among solid phase fractions of studied soils as shown in Fig. 5. For instance, nWTRs addition to clayey soil at rates of 0.1 and 0.3% decreased As occupancy in the exchangeable fraction from 5.4 to 2.5% and 0.03, respectively, whereas in sandy soil, the corresponding changes were from 9.9 to 2.9% and 0.04, respectively. Generally, this exchangeable fraction is easily available for plant uptake since simple change in soil solution ionic strength can cause metal releasing (Coquery & Wekbourn, 1999; Wan et al., 2017). Similarly, the bulk WTRs reduced As percentage in exchangeable fraction to a lesser extent than nWTR. Thus, application of nWTRs to contaminated soils can significantly contribute in reducing the bioavailable form of As in soil media. Furthermore, the residual fraction (RS) was strongly affected by nWTRs application. At 0.3% nWTRs application, the RS (immobile) fraction noticeably increased from 19.80 and 48.51% to 88.75 and 85.58%, for clayey and sandy soils, respectively. Introducing the nanoscale WTRs at higher rate clearly governed As mobility in both As-contaminated soils. In alkaline soils, the presence of Fe/Al, Ca/Mg and/or organic/sulfides can be an important mechanism for As immobilization by forming new As phases precipitate (Cheng et al., 2005; Datta et al., 2007). Rahman et al. (2017) demonstrated that formation of Ca-, Al- and Fe-arsenates precipitates has led to conversion of As from monodentate–mononuclear to bidentate–binuclear sites. Our results indicate incontestably that nWTRs applications have substantial impact on limiting the more labile fractions in As-contaminated soils. In brief, application of nWTRs decreased As occupancy in the exchangeable fraction in clayey and sandy soil and greatly increased the association of As with the residual fraction and in turn enhanced As immobilization in studied soils. Such changes are likely due to changes from external to interlayer adsorption process which suggested long-term As stability**.**

## Arsenic speciation

Arsenic species in soil strongly affects bioavailability and mobility of this element in the environment (Arai et al., 2006; Kim et al., 2014; Niazi et al., 2011; Smith et al., 2009). The influence of WTRs**/**nWTRs application on As species in the soil solution of clayey and sandy soils solution was studied. Arsenious acid (H_3_AsO_3_) was the dominant arsenic species (> 90%) `in both un-amended soils with very low percentage of oxyanions arsenate (AsV) species (Table 5). The arsenious acid represents ~ 91% of the total As present in soil solution of both control (un-amended) soils. In natural aqueous media with pH ranged from 4 to 10, most As(III) and As(V) species exist in H_3_AsO_3_, H_2_AsO^−^_4_ and HAsO^−2^_4_ forms (Bissen & Frimmel, 2003; Fendorf & Kocar, 2009) and As(V) could be converted to As(III) by microorganisms action (Gallagher et al., 2001). The high percentage of uncharged arsenite forms in both studied soils indicates potential increase in mobility and toxicity of As in soil environment due to the low sorption affinity of this electrically neutral form toward minerals surfaces when the pH is below 9 (Wu et al., 2015).

**Table 5:** Arsenic species percentage of total in the soil solution of contaminated soils amended with WTRs or nWTRs

Species (%)	0WTR	2%WTRs	0.1% nWTRs	0.3% nWTRs
*Clay soil *				
H_2_AsO^−^_4_	1.01	1.98	3.86	3.98
H_3_AsO_3_	91.71	57.51	53.83	45.59
HAsO^−2^_4_	0.23	1.65	2.53	3.76
As_2_O_3_	3.63	3.00	2.63	1.76
As(OH)_3_	2.86	2.87	3.92	5.54
As(OH)_5_	0.56	32.99	33.23	39.37
*Sandy soil *				
H_2_AsO^−^_4_	0.11	2.81	3.10	4.61
H_3_AsO_3_	90.69	49.32	47.47	40.60
HAsO^−2^_4_	0.00	0.59	1.92	2.90
As_2_O_3_	2.91	1.99	1.16	0.99
As(OH)_3_	3.61	3.98	3.99	4.98
As(OH)_5_	2.68	41.31	42.36	45.92

The addition of nWTRs and WTRs to both soils strongly influenced As species in soils solution, and this effect was more manifested at high application rate of nWTRs. For instance, nWTRs application at a high rate of 0.3% extremely diminished the percentage of arsenious acid in solutions from 91.71 to 45.59% and from 90.69 to 40.60% for clayey and sandy soils, respectively. Furthermore, application of nWTRs at different rates to both soils markedly increased the percentage of the less toxic amorphous arsenic hydroxide (As(OH)_5_) species (Table 5). Thus, the overall findings highlight the role of nWTRs in reducing the mobility and toxicity of As in soil environment.

## FTIR spectra and mechanism of As sorption

**Fig. 6:**
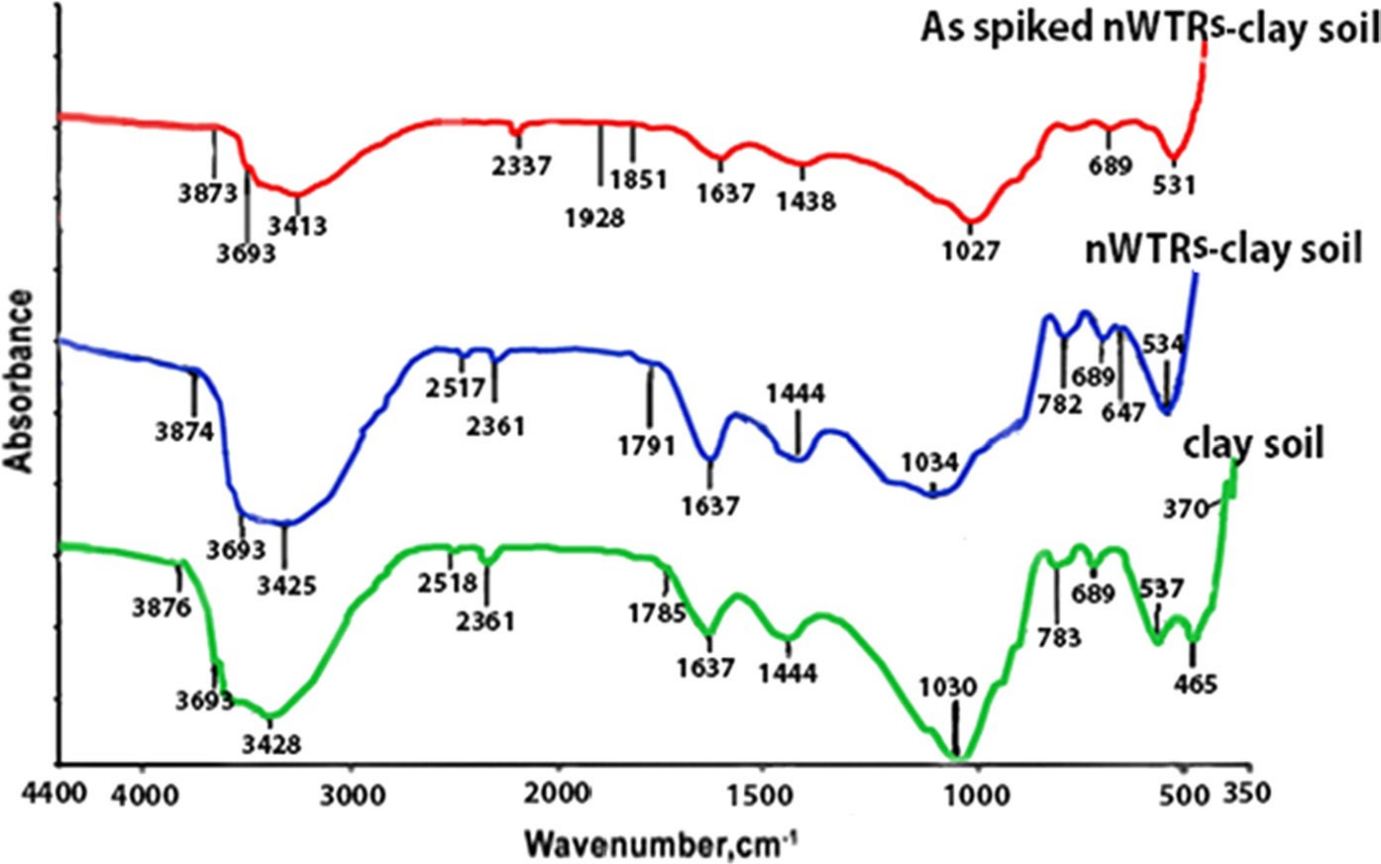
Fourier transmission infrared spectrum of clay soil, nWTRs clayey soil and As-spiked nWTRs clay soil. nWTRs, water treatment residual nanoparticles

A Fourier infrared spectroscopy study was performed to further investigate the mechanism of As sorption by nWTRs-amended soils. The spectrum of clayey soil prior/after application of nWTRs is shown in Fig. 6. The FTIR spectra of the un-amended clayey soil showed two adsorption bands corresponding to HOH at 3428 and 1637 cm^−1^ attributed to stretching vibration of the hydrogen bonded OH groups and bending vibration of the free water molecules, respectively. In addition, various bands appeared at 2518, 1444, 1030, 783, 689, 537 and 465 cm^−1^ are referred to OH stretching of carboxylic group, stretching vibration of CO_3_^2−^, Si–O groups stretching vibrations, the bending vibrations of Al–O, Al–OH, Al–O–Si and Si–O–Si groups, respectively (Blanch et al., 2008; Janik et al., 2007; Kim et al., 2004; Madejová, 2003). Changes in intensity and wave number of vibrations bands were observed as a result of amending clayey soil with nWTRs. As seen in Fig. 6, the stretching vibration band of Si–O at 1030 cm^−1^ has been broadened and Si–O–Si vibration band at 465 cm^−1^ has been disappeared. Also, the oscillation band at 537 cm^−1^ related to Al–O–Si has been changed toward lower wave number. A shift in OH stretching band at 3428 cm^−1^ toward lower frequency is also observed. These changes indicate the interaction between nWTRs and clayey soil. Moreover, the high reduction in frequency and strength of the OH stretching band at 3413 cm^−1^ in the spectra of As-contaminated clayey soil amended with nWTRs indicates the strong interaction between As and OH group of nWTRs. The disappearance of OH stretching belongs to COOH group at 2517 cm^−1^ and also confirms the involvement of OH group in As adsorption reaction, and the decrease in the strength of HOH bending vibration at 1637 cm^−1^ after As adsorption insures interaction between As- and nWTRs-treated clayey soil (Bermudez, 2010; Tarte, 1967).

**Fig. 7:**
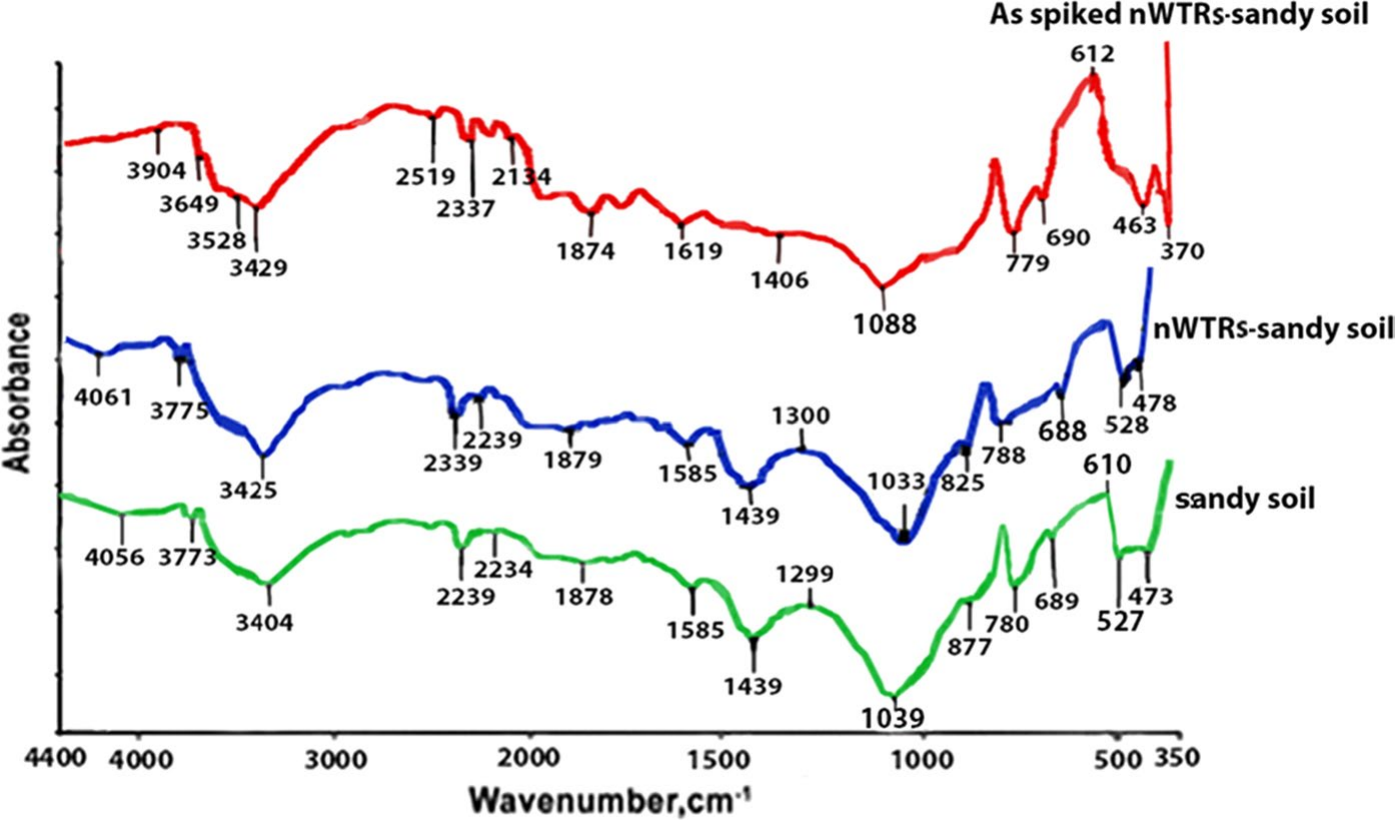
Fourier transmission infrared spectrum of sandy soil, nWTRs- sandy soil and As-spiked nWTRs sandy soil. nWTRs, water treatment residual nanoparticles

The FTIR spectra of the sandy soil are presented in Fig. 7. The spectrum shows peaks at 3773 and 3404 cm^−1^ that corresponding to OH stretching free and OH stretching H-bonded, respectively. Different peaks appeared at wave numbers of 1439, 1039, 877, 780, 527 and 473 cm^−1^ are related to γ-Al_2_O_3_, CO_3_^2−^ stretching vibration, bending vibration of Fe–OH, quartz mixture, Al–O–Si and Si–O–Si bending vibrations, respectively (Blanch et al., 2008; Janik et al., 2007; Kim et al., 2004; Madejova, 2003; Yang et al., 2018). After amending sandy soil with nWTRs, the OH stretching H-bonded at 3404 cm^−1^ has been strongly shifted toward higher frequency at 3425 cm^−1^. Also, there was a strong shift in the bending vibration of Fe–OH at 877 to 825 cm^−1^. Furthermore, the Si–O–Si at 473 cm^−1^ was changed to higher wave number. These changes in the peaks frequencies indicate nWTRs attraction toward colloid soil surfaces. The changes of FTIR spectrum of As-spiked sandy soil amended with nWTRs were as follows: (1) The OH stretching free band at 3775 cm^−1^ was moved to lower wave number at 3649 cm^−1^ which demonstrated the involvement of the OH group in the As interaction. (2) Appearance of Fe–OH asymmetric stretch at 463 cm^−1^ suggested the contribution of Fe–OH of nWTRs in the As interaction through the OH group. (3) The Si–O–Si bending vibrations band (478 cm^−1^) has been increased in intensity and switched to lower wave number (463 cm^−1^) which indicates the interaction between Si–O–Si of nWTRs and As. (4) The Al–O–Si band at 528 cm^−1^ has been vanished designating its charring in As sorption. (5) The γ-Al_2_O_3_ at 1439 cm^−1^ has been reduced in strength and shifted to lower wave number. The aforementioned findings suggest different reaction mechanisms taking place between As and the surfaces of amorphous Fe and Al oxides of nWTRs through OH group. A number of studies have been executed to investigate in depth the surface complexation mechanisms of As species on pure Fe and Al hydro-oxides in addition to Fe/Al-WTRs using X-ray absorption spectroscopy tool (Ladeira et al., 2001; Makris et al., 2007; Sherman & Randall, 2003).

**Fig. 8:**
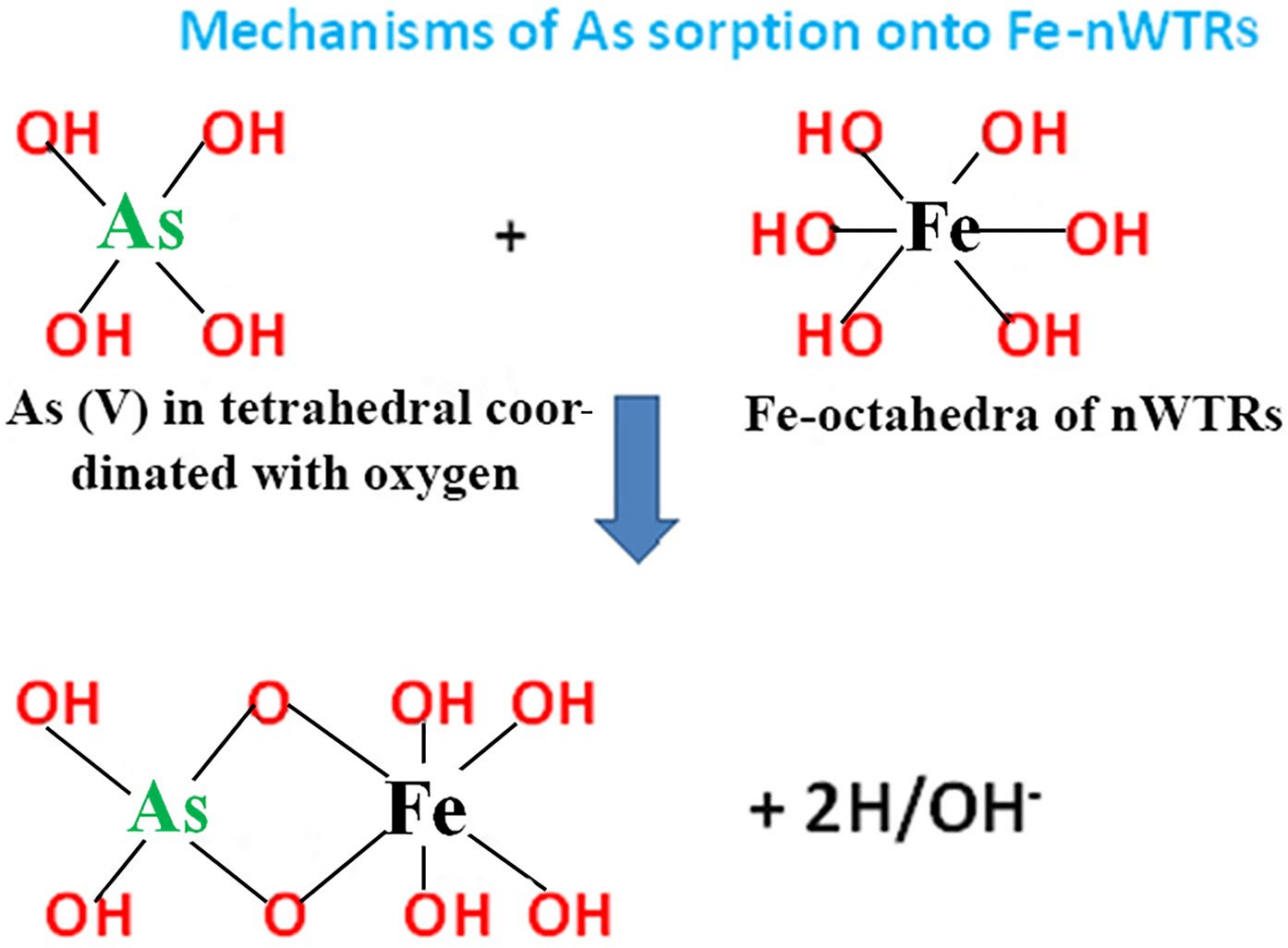
Schematic diagrams of surface complexation of arsenate onto Fe-nWTRs resulting a bidentate reaction between AsO_4_ of arsenate and one adsorption site of Fe

**Fig. 9:**
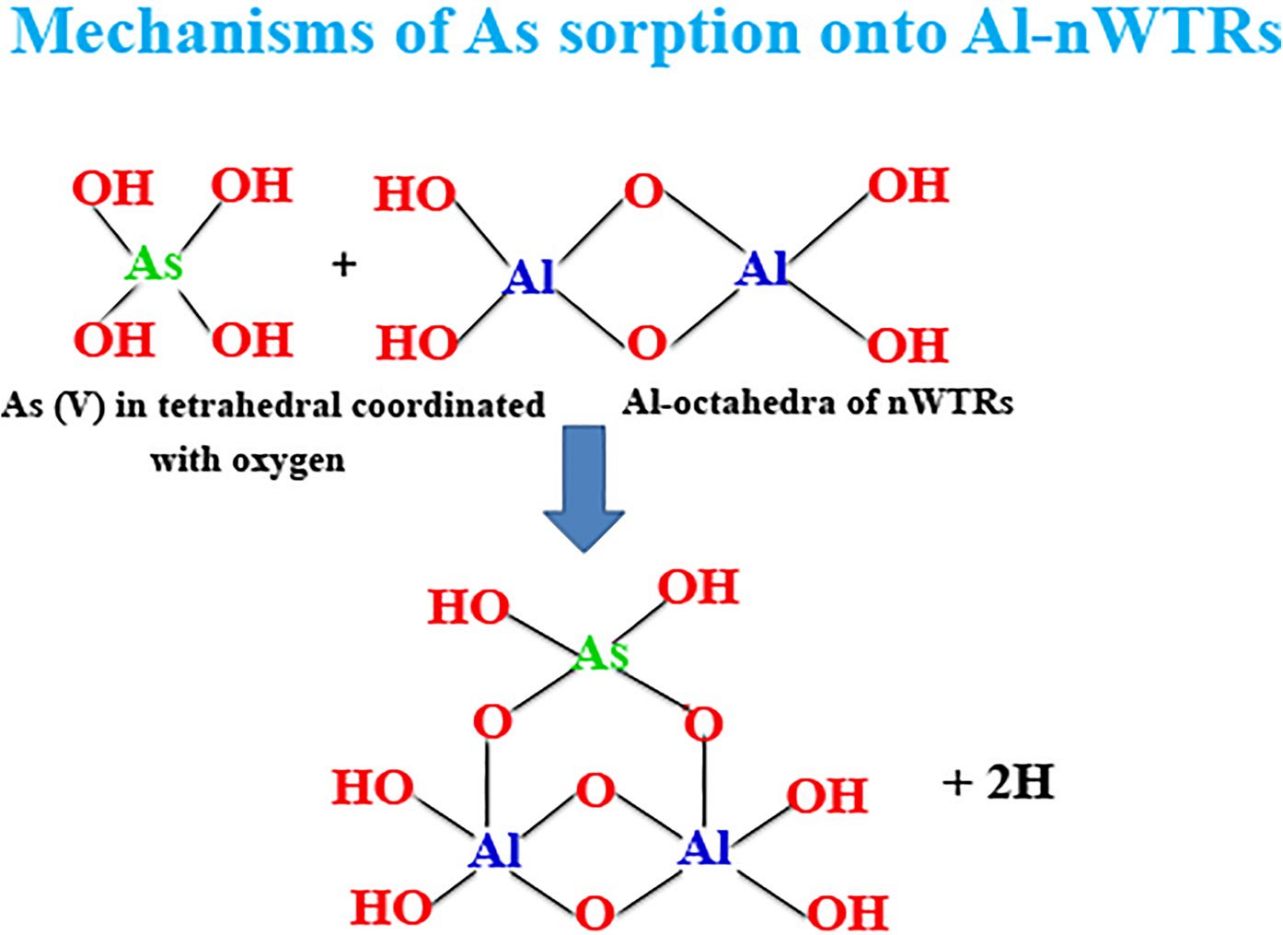
Schematic diagrams of surface complexation of arsenate onto Al-nWTRs resulting a bidentate reaction between AsO_4_ of arsenate and two adsorption site of Al

These studies concluded that both Fe/Al oxides and Fe/Al-WTRs can form inner-sphere surface complexes with arsenate and arsenite with some structural mechanism difference between Fe/Al oxides and Fe/Al-WTRs due to the heterogeneous nature of WTRs. The nanostructured nWTRs simultaneously contain high percentage of Fe/Al oxides, along with other substances such as humic materials associated with minerals (Makris et al., 2007). Therefore, our study suggests bidentate mononuclear and bidentate binuclear inner-sphere complexes between arsenate onto Fe-nWTRs and Al-nWTRs, respectively, as illustrated in Figs. 8 and 9.

## A comparison between previous studies dealing with As stabilization in contaminated soils and current study

Table 6 presents the studies dealing with Arsenic stabilization in contaminated soils using different materials. The data presented in Table 6 clearly demonstrated the high capability of nWTR to immobilize and stabilize As in contaminated soils.

**Table 6:** A comparison between previous studies dealing with As stabilization in contaminated soils and current study

Materials	Treatment (wt%)	Effect	References
Nanowater treatment residuals (nWTRs)	0.3%	Strongly increased soils retention capacity for As and accelerate its sorption rate. The non-residual As fractions dramatically decreased from 80.2 and 51.49% to 11.25 and 14.42% in clayey and sandy soils, respectively	Current study
Manganese ferrite nanoparticles	1 and 10%	Decrease in arsenite leachability/availability, association of Fe–Mn oxide bounds increased to 70.2 and 82.3%	Zialame et al., (2021)
Granular ferric hydroxide [Fe(OH)_3_], mine sludge containing goethite	5% 5%	Reduced the 30 and 50% of leaching when using mine sludge and Fe(OH)_3_, respectively	Ko et al., (2012)
Compost, zerovalent iron grit [Fe(0)], coal fly ash (CZA)	5% 2% 5%	Decreased total concentrations through leaching in the long term decreased exchangeable fraction and fraction associated with poorly crystalline Fe oxides increased residual fraction	Kumpiene et al., (2012)
Drinking water treatment residuals (WTR)	2.5, 5, and 10%	Fe-WTR and Al-WTR were able to reduce soil As bioaccessibility and phytoavailability	Sarkar et al., (2007b)

## Conclusions

The applicability of water treatment residual nanoparticles (nWTRs) to clayey and sandy contaminated alkaline soils was evaluated for its efficacy in reducing arsenic mobility in soil environment. Adsorption isotherms and kinetics data of As by nWTRs-amended soils best fitted to Langmuir and second-order/power function models, respectively. Amending both soils with bulk and nanoparticles of WTRs increased soils retaining capability for As with nanoparticles being the most effective. Thus, novel application of nWTRs can be more cost-efficient and environmentally friendly compared to conventional treatment techniques. Applying higher rates of nWTRs to both soils markedly increased the percentage of the less toxic amorphous arsenic hydroxide (As(OH)_5_) species as well as the residual (immobile) fraction in As-contaminated clayey and sandy soils. However, further studies calibrating the critical application rate of nWTRs in As-contaminated soils for optimum plant growth and decreased phytoavailability of As are required.

## Supplementary Information

Below is the link to the electronic supplementary material.

Supplementary file1 (XLS 145 kb)

Supplementary file2 (XLS 143 kb)

Supplementary file3 (DOCX 1086 kb)

## Data Availability

All data generated or analyzed during this study are included in this published article and its supplementary information files.
