# Supplementary material for: Potential use of nanoparticles produced from byproducts of drinking water industry in stabilizing arsenic in alkaline-contaminated soils
Source: Environ Geochem Health. 2023 Jun 28;45(8):6727–43. doi: 10.1007/s10653-023-01663-z (PMC10403416; doi:10.1007/s10653-023-01663-z)
Supplement: Supplementary file 3 — Supplementary file3 (DOCX 1086 kb) [file 10653_2023_1663_MOESM3_ESM.docx]

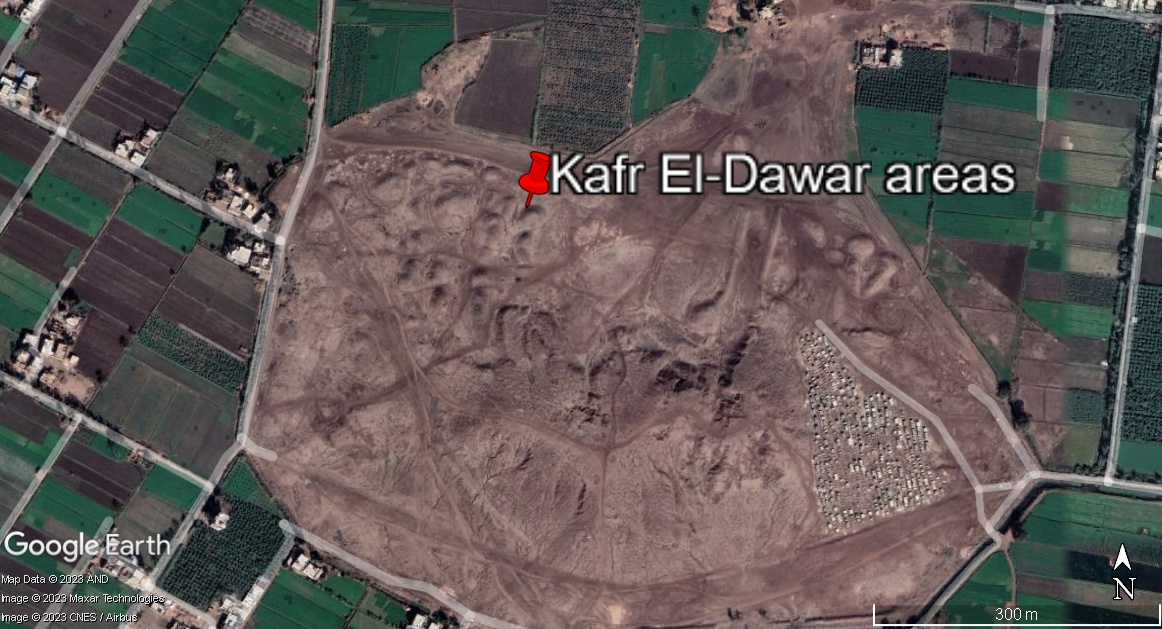


Fig: S1. Kafr Al Dawwar district , Beheira Governorate , Egypt (31º 13′ N, 30º 25′ E ).


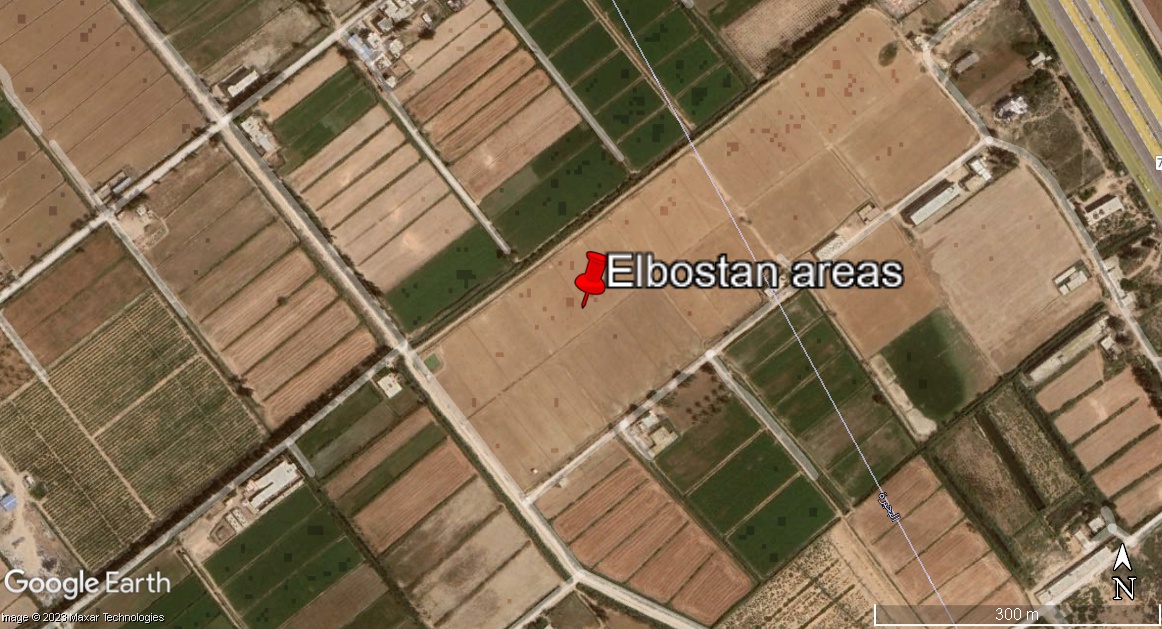


Fig: S2. Elbostan, Alexandria, Egypt (30º 54′ N, 29º 52′ E).


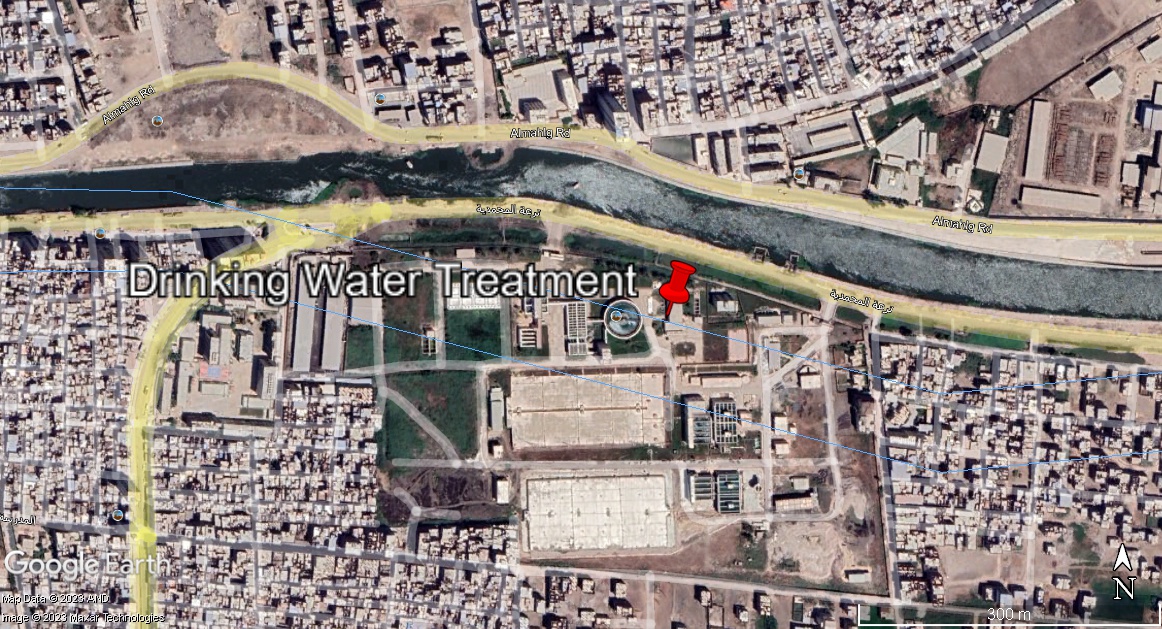


Fig: S3. Kafr El-Dawar drinking water treatment facilities (31º 08′ N, 30º 08′ E).

Table S1. Elovich and Temkin isotherms parameters for As sorption onto the studied soils as affected by WTRs/nWTRs treatments.

| Treatment | Elovich  $\frac{q_{e}}{q_{m}}=K_{E}C_{e}\exp(-\frac{q_{e}}{q_{m}})$ | | | |  | Temkin  $\theta=\frac{RT}{\Delta Q}lnK_{0}C_{e}$ | | | |
| --- | --- | --- | --- | --- | --- | --- | --- | --- | --- |
|  | K_E_  (L mg^−1^) | q_max_  (μg g^−1^) | R^2^ | SE |  | ∆Q  (kJ mol^−1^) | K_0_  (L mg^−1^) | R^2^ | SE |
|  | Clayey soil | | | | | | | | |
| 0WTRs | 0.0064 | 1111.11 | 0.515 | 0.3373 |  | 1.359 | 0.196 | 0.848 | 0.9718 |
| 2%WTRs | 0.0323 | 1111.11 | 0.818 | 0.2718 |  | 4.147 | 0.523 | 0.776 | 0.2957 |
| 0.1%nWTRs | 0.0348 | 909.091 | 0.832 | 0.3041 |  | 3.955 | 0.477 | 0.739 | 0.3148 |
| 0.2% nWTRs | 0.0531 | 1428.57 | 0.934 | 0.1101 |  | 10.229 | 1.129 | 0.700 | 0.1749 |
| 0.3% nWTRs | 0.338 | 909.091 | 0.872 | 0.2746 |  | 18.115 | 6.087 | 0.419 | 0.1509 |
|  | Sandy soil | | | | | | | | |
| 0WTRs | 0.0029 | 714.286 | 0.634 | 0.1768 |  | 2.268 | 0.138 | 0.749 | 0.8916 |
| 2%WTRs | 0.0079 | 400 | 0.739 | 0.8666 |  | 1.233 | 0.149 | 0.521 | 1.3837 |
| 0.1%nWTRs | 0.0102 | 434.783 | 0.793 | 0.7085 |  | 1.243 | 0.166 | 0.496 | 1.3404 |
| 0.2% nWTRs | 0.0212 | 909.091 | 0.838 | 0.3009 |  | 3.822 | 0.477 | 0.459 | 0.76192 |
| 0.3% nWTRs | 0.0300 | 5000 | 0.548 | 0.1356 |  | 8.641 | 2.137 | 0.756 | 0.21931 |

R^2^, determination coefficient; SE, standard error of estimate; WTRs, water treatment residuals; nWTRs, water treatment residual nanoparticles.
